# PAN-cODE: COVID-19 forecasting using conditional latent ODEs

**DOI:** 10.1093/jamia/ocac160

**Published:** 2022-09-01

**Authors:** Ruian Shi, Haoran Zhang, Quaid Morris

**Affiliations:** Department of Computer Science, University of Toronto, Toronto, Canada; Vector Institute for Artificial Intelligence, Toronto, Ontario, Canada; Department of Computer Science, University of Toronto, Toronto, Canada; Vector Institute for Artificial Intelligence, Toronto, Ontario, Canada; Department of Computer Science, University of Toronto, Toronto, Canada; Vector Institute for Artificial Intelligence, Toronto, Ontario, Canada; Computational and Systems Biology, Memorial Sloan Kettering Cancer Center, New York City, New York, USA

**Keywords:** pandemic prediction, deep learning, time series forecasting, latent variable models

## Abstract

The coronavirus disease 2019 (COVID-19) pandemic has caused millions of deaths around the world and revealed the need for data-driven models of pandemic spread. Accurate pandemic caseload forecasting allows informed policy decisions on the adoption of non-pharmaceutical interventions (NPIs) to reduce disease transmission. Using COVID-19 as an example, we present Pandemic conditional Ordinary Differential Equation (PAN-cODE), a deep learning method to forecast daily increases in pandemic infections and deaths. By using a deep conditional latent variable model, PAN-cODE can generate alternative caseload trajectories based on alternate adoptions of NPIs, allowing stakeholders to make policy decisions in an informed manner. PAN-cODE also allows caseload estimation for regions that are unseen during model training. We demonstrate that, despite using less detailed data and having fully automated training, PAN-cODE’s performance is comparable to state-of-the-art methods on 4-week-ahead and 6-week-ahead forecasting. Finally, we highlight the ability of PAN-cODE to generate realistic alternative outcome trajectories on select US regions.

## INTRODUCTION

**Figure 1.**
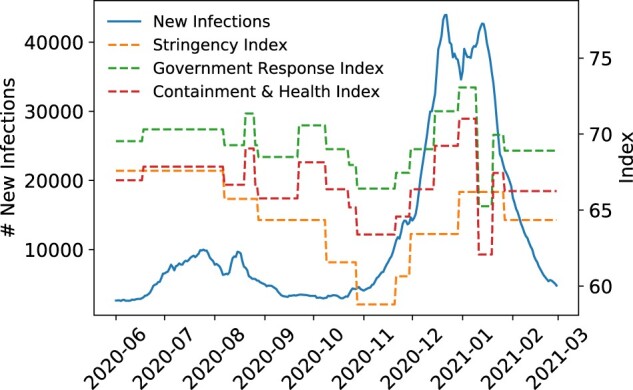
The number of daily new infections over time in the state of California plotted alongside indices representing the level of NPIs adopted. These indices are provided by the Oxford COVID-19 Government Response Tracker^34^ and summarize the level of NPIs adopted into a single metric scored out of 100. NPIs have a time-delayed, negatively correlated effect on COVID-19 caseload, seen most clearly from 2020–2010 to 2021–2001.

The coronavirus disease 2019 (COVID-19) pandemic remains a severe threat to public health, affecting 235 million individuals, and causing 4.8 million deaths as of October 2021.^1^ Forecasting future disease caseload is critical for pandemic response^2^ and enables informed policy decisions on medical resource allocation and policies to reduce disease transmission. For COVID-19, non-pharmaceutical interventions (NPIs) such as social distancing and mask mandates are critical in slowing transmission^3^ and remain important even as vaccines are deployed.^4^ In Figure 1, we visualize the negatively correlated relationship between NPI adoption and COVID-19 transmission in the state of California. While NPIs drastically reduce COVID-19 transmission, they also incur high socio-economic costs,^5^^,^^6^ motivating the ability to model future COVID-19 caseload as a function of NPI adoption stringency. Current approaches to caseload forecasting include statistical methods,^7^^,^^8^ compartmental models,^9–14^ deep neural networks,^15–20^ or ensembles such as the COVID-19 Forecast Hub.^21^ These methods provide reliable short-term forecasting, but as we further outline in Supplementary Appendix A, many do not offer predictions past 4 weeks, cannot explicitly model the relationship between NPI adoption and future caseload, and require expert-intervention to be fit to data.

Here, we introduce the Pandemic conditional Ordinary Differential Equation (PAN-cODE), a fully-automated pandemic caseload forecasting method capable of conditioning forecasts on the stringency of NPI adoption. PAN-cODE uses deep neural networks in a Latent Ordinary Differential Equation (ODE)^22^ architecture to learn a conditional generative model of caseload dynamics only using past daily infection/death counts and NPI policy. When trained on US state and county level COVID-19 caseload data,^23^ PAN-cODE offers 4-week forecasts with accuracy comparable to state-of-the-art methods and beats all COVID-19 Forecast Hub methods at 6-week-ahead forecasting while using minimal data features. We show that PAN-cODE can generalize learned dynamics to accurately predict outcomes in unseen regions. Finally, we demonstrate PAN-cODE’s ability to generate realistic outcomes based on alternative NPI adoptions for selected US states.

## BACKGROUND

### Deep epidemiological forecasting

Epidemiological forecasting is traditionally performed using compartmental SIR models^24–26^ or time series methods.^7^^,^^8^ By leveraging the non-linear representational power of deep neural networks, deep learning approaches for epidemiological forecasting aim to better capture the complex relationship between historical caseloads, high-dimensional covariate time series, and future caseload trajectories. Current deep epidemiological forecasting methods use neural networks to estimate compartmental model coefficients from input features,^9^^,^^15^ integrate spatio-temporal graph neural networks to model how geographic proximity affects viral transmission,^20^^,^^27–30^ or apply deep time series methods such as LSTMs^14^^,^^18^^,^^19^ for forecasting.

### Neural and latent ODEs

PAN-cODE learns COVID-19 caseload dynamics from historical data using the Neural ODE.^31^ Where $x_{0:N}\;$denotes input data and $t_{0:N}\;$represents their time of observation, the Neural ODE represents time series as the solution to an ODE such that:

$$x_{0:N}=\mathrm{ODESolve}(f_{\theta },x_{0},\;t_{0:N})$$

where $f_{\theta }$ represents $\frac{dx}{dt}=f(x,\;t,\;\theta )$ and is parameterized by a neural network. Alternatively, $f_{\theta }$ could be parameterized by a known ODE, such as the SIR equations, where unknown parameters are then optimized by the Neural ODE through backpropagation.

The Neural ODE can be arranged into a variational auto-encoder architecture known as the Latent ODE^22^ to increase its representational ability. The Latent ODE first encodes multidimensional input features using the GRU-ODE: a Gated Recurrent Unit (GRU)^32^ that evolves hidden states between observations using a Neural ODE. The GRU-ODE encoder outputs the parameters for a factorized Gaussian variational distribution over the initial state of a latent trajectory. From this variational distribution, we sample a latent initial state denoted z0, and solve a separate Neural ODE parameterized by $f_{\psi }$ from z0 to obtain a latent trajectory. Finally, this latent trajectory is decoded with neural network $f_{\phi }$ into the output trajectory. The Latent ODE is represented by the following set of equations:

$$\begin{array}{l} \mu_{z0},\;\sigma_{z0}^{2}={\text{GRUODE}}_{f_{\theta }}\left(x_{0:N},\;t_{0:N}\right)\\ \;z0\;∼\;q(z0\;|\;x_{0:N})=N\left(\mu_{z0},\;\sigma_{z0}^{2}\right)\\ \;z_{0:N}=\text{ODESolve}\left(f_{\psi },\;z0,\;t_{0:N}\right)\\ \;x_{i}\;∼\;N\left(f_{\phi }\left(z_{i}\right),\;\sigma^{2}\right)\;\;\;\text{for}\;i=1,\;\ldots ,\;N \end{array}$$

where $\sigma^{2}$ is a fixed variance term. The Latent ODE is trained by maximizing the evidence lower bound (ELBO), defined as:

$$E_{z0\;∼\;q(z0\;|\;x_{0:N})}\left[\text{log}\;p\left(x_{0:N}\right)\right]-\text{KL}\left[q(z0\;|\;x_{0:N})∥p\left(z0\right)\right]$$

## METHOD

**Figure 2.**
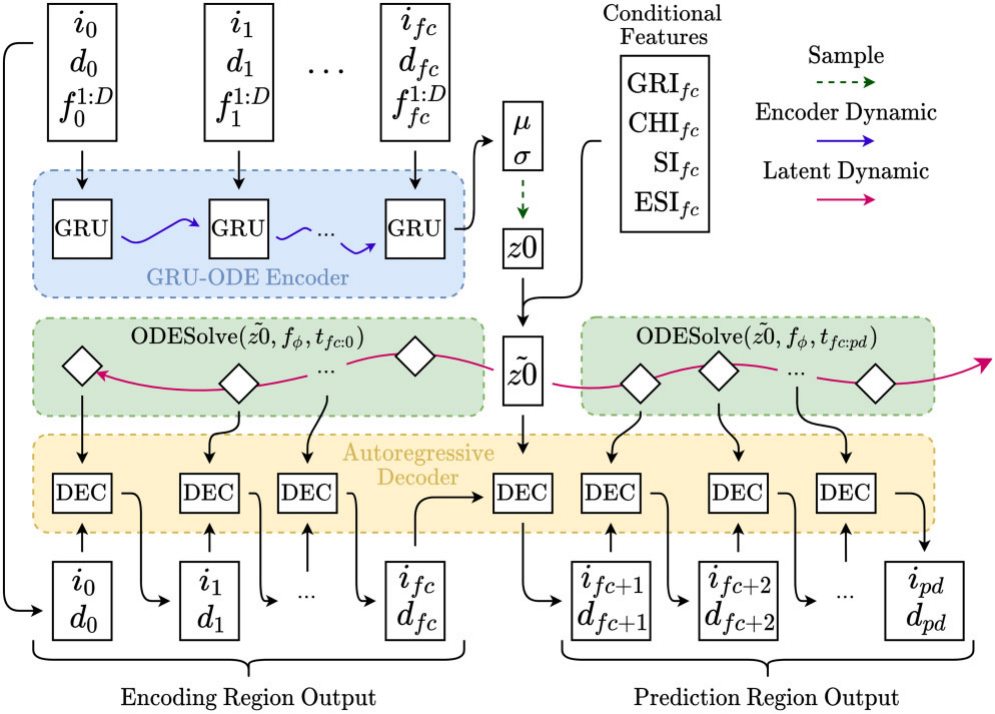
Architectural schematic of the PAN-cODE. We input the daily counts of infection (i), death (d), and other covariates $\left(f^{1:D}\right)$ up to forecast date fc. The encoding region time series are encoded by a GRU-ODE encoder, which outputs parameters $\mu_{z0},\;\sigma_{z0}^{2}$ for the variational posterior on the latent initial state. We obtain a sample latent initial state z0 from the variational posterior, which is then concatenated with the conditional features to obtain the augmented latent initial state $\tilde{z0}$. From $\tilde{z0}$, the Neural ODE is used to solve the latent trajectory for the prediction and encoding regions, which is passed through the autoregressive decoder to output the predicted daily infection and death count, up to prediction date pd.

PAN-cODE introduces novel modifications to the Latent ODE architecture to allow generation of alternative forecasts conditioned on the stringency of NPI adoption. We visualize the PAN-cODE architecture in Figure 2. PAN-cODE first learns a representation of current caseload dynamics using observed data in the Encoding Region, which includes input data up to the forecasting date, denoted fc. The objective is to output the caseload in the Prediction Region, which begins on the forecasting date and extends to the prediction date, denoted pd. We denote the encoding region daily infection and death count as $i_{0:fc}$ and $d_{0:fc}$, respectively. Other covariates, denoted $f_{0:fc}^{1:D}$, are also included as input for the GRU-ODE encoder. We assume regularly spaced time intervals between observations and arbitrarily set $t_{0:fc}$. However, in contrast to most other time-series prediction methods, irregularly sampled time series can be easily handled by setting $t_{0:fc}$ to the actual times of observation. The GRU-ODE encoder outputs the parameters ($\mu_{z0},\;\sigma_{z0}^{2})$ for the variational distribution over the initial state of a latent trajectory.

PAN-cODE introduces the conditional Latent ODE, which extends work on deep conditional generative models^33^ to the Latent ODE. The conditional Latent ODE allows the latent initial state to be conditioned on injected data features. For PAN-cODE, we condition on 4 metrics representing the stringency of NPI adoption at forecasting time, denoted $I_{fc}\in R^{4}$. These metrics are described in the experimental section. The conditional Latent ODE is implemented by concatenating the vector of conditional features to the sampled latent initial state. Letting $X=\left[i_{0:fc},\;d_{0:fc},\;f_{0:fc}^{1:D}\right]$, the augmented latent initial state $\tilde{z0}$ is obtained as:

$$\begin{array}{l} \mu_{z0},\;\sigma_{z0}^{2}={\text{GRUODE}}_{f_{\theta }}\left(X,\;t_{0:fc}\right)\\ z0\;∼\;N\left(\mu_{z0},\;\sigma_{z0}^{2}\right)\\ \;\tilde{z0}=\left[z0,\;I_{fc}\right] \end{array}$$

Consequently, the ELBO objective becomes:

$$E\left[\log p\left(x_{0:N}\right|\;I_{fc})\right]-\mathrm{KL}\left[q\left(z0\;\right|\;x_{0:N},\;I_{fc})∥p\left(z0|I_{fc}\right)\right]$$

During training, the model learns a correlational relationship between the conditional variables and caseload forecasts. Afterwards, alternative caseload trajectories can be generated by modifying the values of $I_{fc}$ to correspond to modified NPI adoption stringency.

PAN-cODE uses an auto-regressive decoder (ARD) to convert latent trajectories into data space. The ARD is a simple feed-forward neural network combining the latent output at time t with the previously predicted output at time t-1, thus representing predictions as a function of the previous output. This is represented in the equation:

$$\left(i_{t},\;d_{t}\right)=\mathrm{Linear}\left(i_{t-1},\;d_{t-1},\;z_{t}\;|\;\phi \right)$$

where parameters ϕ are shared across timepoints. The ARD serves to restrict the maximum change in caseload between timepoints when mapping to the data space.

PAN-cODE is trained to reconstruct Encoding Region observations and make predictions in the Prediction Region. After obtaining augmented latent initial state $\tilde{z0}$, PAN-cODE obtains Encoding Region reconstructions by solving a latent trajectory backwards in time from the forecast date to the initial date and decoding it using the ARD:

$$\left(i_{fc:1},\;d_{fc:1}\right)=\mathrm{ARD}\left(\mathrm{ODESolve}\left(\tilde{z0},\;f_{\phi },\;t_{fc:1}\right)\right)$$

Next, a latent trajectory is solved forward in time to obtain the caseload trajectory in the Prediction Region:

$$\left(i_{fc:pd},\;d_{fc:pd}\right)=\mathrm{ARD}\left(\mathrm{ODESolve}\left(\tilde{z0},\;f_{\phi },\;t_{fc:pd}\right)\right)$$

Forecasts for longer prediction windows can be obtained by simply modifying pd.

## EXPERIMENTS

### Data

We train PAN-cODE using daily COVID-19 caseload counts in US states and counties from Google Cloud Platform’s (GCP) Open Data resource.^23^ Roughly 2500 trajectories are available, each with daily observations since February 2020. We apply a 7-day rolling average function to remove reporting noise and apply a shifted log transform of $\log\left(x+1\right)$ for numerical stability.

We provide PAN-cODE with historical NPI adoption stringency as tracked by the Oxford COVID-19 Government Response Tracker.^34^ The daily status of NPI adoption is represented by a vector of integers denoting the level of adoption for a specific NPI. When forecasting deaths, we add a 14-day delay between these features and COVID-19 caseload to account for the delayed effect of social restrictions.^35^ We also include the daily temperate index from GCP to account for seasonality in COVID-19. The choice of data smoothing function and feature shift value was selected experimentally and is shown in Supplementary Appendix C.

We use 4 metrics provided by OxGRCT as the conditional features in PAN-cODE. These metrics aggregate the level of NPI adoption in specific areas into a scalar score out of 100, where a higher score indicates higher stringency. These metrics are the Stringency Index, the Government Response Index (GRI), the Containment Health Index (CHI), and Economic Support Index. We standardize these metrics and use their values on the forecasting date as the conditional latent variables. These indices are also used as input covariates.

### Hyper-parameters and training

We randomly vary the forecasting date per training epoch to expose PAN-cODE to the correlation between conditional features and future caseloads under various conditions. We first set a validation cut-off date such that data after this date are unseen during training. For each training epoch, we randomly sample an epoch forecast date from between the start date and the validation cut-off date. PAN-cODE is provided data up the epoch forecast date and is tasked with predicting caseload 4 weeks ahead. PAN-cODE is trained by maximizing the ELBO using the Adam optimizer.^36^ We describe hyper-parameters in Supplementary Appendix D.

### Tasks and evaluation

We evaluate the forecasting ability of the PAN-cODE using prediction windows of 4 and 6 weeks. We evaluate against baseline methods using the metrics of median absolute error (MAE) and mean rank, which is computed as the average, across the 51-state level regions, of the rank of the method’s MAE in ranked list of the MAE’s of all compared methods. We include an evaluation on data from regions unseen during training to highlight the generalization performance of PAN-cODE. We generate forecasts for up to 6 weeks from 2 forecasting dates: December 28, 2020 and March 8, 2021 to evaluate the model’s performance under different stages of COVID-19 progression. Data after each forecasting date are not used during training or model selection. The source code for PAN-cODE is available at: https://github.com/morrislab/PAN-cODE.

## RESULTS

### PAN-cODE outperforms baselines on 6-week forecasts

In Table 1, we compare the performance of PAN-cODE on forecasting the number of deaths in US states against our baselines and competitive methods from the COVID-19 Forecast Hub, described in Supplementary Appendix A. We apply the Wilcoxon ranked-sum test using a *P*-value threshold of .05 against the best performing method for each metric and find that the error in PAN-cODE’s forecasts is not significantly larger than that of the best model in every metric. Furthermore, on the March 8, 2021 6-week forecasting task, PAN-cODE provides significantly lower error than all other methods, demonstrating its reliability for long-term forecasting. Daily infection forecasting results are reported in Supplementary Appendix B.

**Table 1.** Median absolute error (MAE) and mean rank of the forecasted total deaths for PAN-cODE versus several baseline methods for all 50 states of the United States and the District of Columbia

	December 28, 2020 forecast				March 8, 2021 forecast			
	4 weeks ahead		6 weeks ahead		4 weeks ahead		6 weeks ahead	
Model	MAE	Mean Rank	MAE	Mean Rank	MAE	Mean Rank	MAE	Mean Rank
**PAN-cODE**	***167***	***7.39***	***207***	***3.63***	***93***	***8.53***	***80***	***4.41***
**JHU_IDD-CovidSP**	210	9.76	***312***	***4.51***	95	11.06	120	5.82
**Covid19Sim-Simulator**	160	***8.43***	***321***	***4.49***	***67***	***9.2***	132	5.31
**IowaStateLW-STEM**	170	***8.88***	***380***	***4.51***	158	13.65	260	7.82
**Columbia_UNC-SurvCon**	210	9.62	446	4.92	108	11.86	145	6.96
**UCLA-SuEIR**	283	12.24	383	5.55	159	13.88	212	8.04
**USC-SI_kJα**	185	8.64	–	–	***84***	10.55	134	5.72
**JHUAPL_Bucky**	432	14.59	853	7.33	***95***	11.18	109	5.96
**GRU-ODE**	371	12.94	644	6.61	***90***	11.39	132	6.1
**VAE-GRU**	964	18.86	1359	9.02	158	13.53	225	7.37
**Google_Harvard-CPF**	***118***	***6.22***	–	–	***86***	***9.49***	–	–
**UMass-MechBayes**	329	11.1	–	–	***74***	***8.12***	–	–
**CU-select**	486	14.2	–	–	***82***	9.96	–	–
**COVIDhub-ensemble**	***154***	***6.91***	–	–	***78***	***8.13***	–	–
**Caltech-CS156**	***156***	***6.10***	–	–	***93***	9.35	–	–
**UCSD_NEU-DeepGLEAM**	225	9.91	–	–	***75***	***8.48***	–	–
**GT-DeepCOVID**	131	8.45	–	–	***79***	***9.48***	–	–
**UA-EpiCovDA**	439	14.98	–	–	***116***	11.69	–	–
**TTU-squider**	259	10.76	–	–	422	17.45	–	–
**Mean COVID Hub**	240	–	380	–	122	–	174	–
**Baseline (PrevWeek)**	218	10.02	343	4.43	120	12.5	295	7.74

*Note*: Mean rank is computed across the 51 ranked lists of methods ordered by increasing MAE. In each column, the best method is underlined; methods whose MAEs are not significantly worse than this model in a non-parametric paired test (ie, Wilcoxon sign-rank *P* > .05) are indicated in bold italics.

### PAN-cODE generalizes to unseen regions

In Table 2, we show the performance of PAN-cODE on countries entirely *unseen* during training. We find that PAN-cODE makes reasonable projections for 4- and 6-week infections forecasts, far outperforming baselines. The VAE-GRU baseline performed extremely poorly in this task and was omitted from our results.

**Table 2.** Percentage error of the incident infections for PAN-cODE versus our baselines on several countries unseen during training, forecasted from December 28, 2020

	4 weeks ahead				6 weeks ahead			
Model	Canada	United Kingdom	India	Russia	Canada	United Kingdom	India	Russia
**Baseline (PrevWeek)**	39.0%	66.7%	63.1%	53.4%	103.4%	213.4%	85.9%	93.0%
**GRU-ODE**	474.4%	36.4%	144.8%	–5.5%	777.7%	156.5%	179.2%	15.4%
**PAN-cODE**	**–10.4%**	**8.2%**	**7.2%**	**0.4%**	**–51.4%**	**–21.6%**	**–55.3%**	**–55.2%**

*Note*: The lowest percentage error is shown in bold.

### PAN-cODE generates sensible alternative forecasts

**Figure 3.**
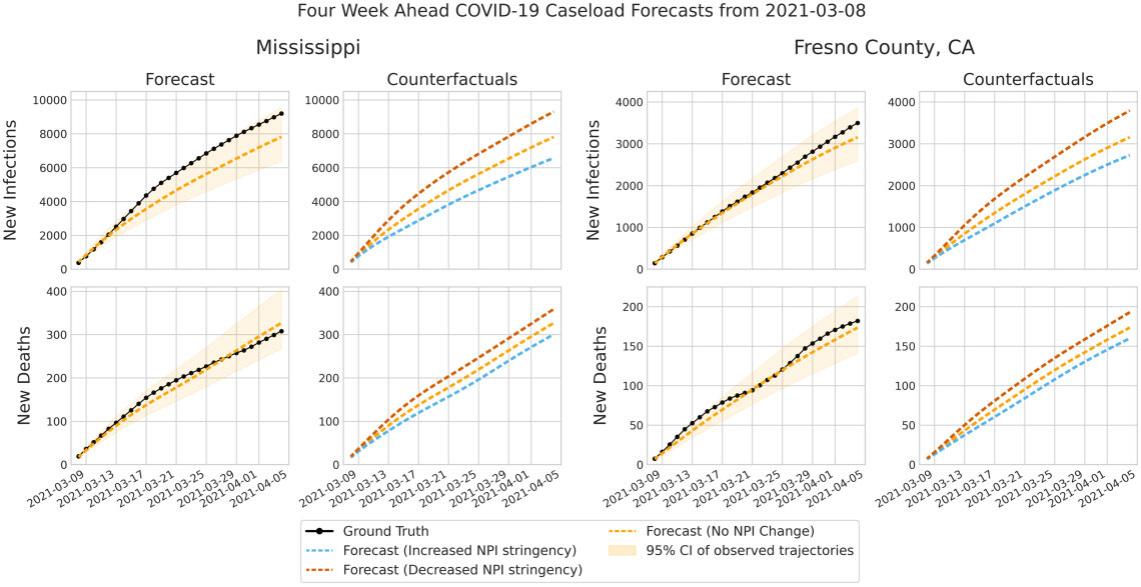
Four week ahead forecasts of COVID-19 infection and deaths at the state (Mississippi) and county (Fresno County, CA) level. The cumulative increase in infections and deaths from the forecast date of May 8, 2021 is shown. PAN-cODE forecasts are conditioned on the OxGCRT Government Response Index, the value of which is modified at the forecasting date to obtain alternative outcome trajectories. We visualize the effects of increasing and decreasing NPI adoption compared to the predicted trajectory, which uses the real NPI adoption magnitude at forecast time. The 95% confidence interval is shown as a shaded area associated with 2 standard deviations from the mean trajectory, as computed by sampling 100 latent trajectories with the modeled observation noise.

In Figure 3, we demonstrate the ability of PAN-cODE to generate alternative outcome trajectories. This ability to generate alternative forecasts can aid policy decisions on NPI adoption for the desired level of transmission reduction. We demonstrate PAN-cODE on both state and county level forecasting and generate alternative trajectories by increasing/decreasing the GRI and CHI measures of NPI stringency. We note that the features for death forecasts are offset by 14 days, meaning the alternative daily death forecasts assume that the change was made 14 days prior.

### The importance of features in forecasting

We computed feature importance estimates using LIME^37^ for the trained PAN-cODE model (see Supplementary Appendix E). The model anticipates that adopting preventative measures has a high impact on infection counts 21 days before the forecast date but that nearer to the forecast date (10 days prior), the actual caseload has the greatest relevance. This behavior is consistent with the known lagged effect of NPIs. Note that different feature importance models make different assumptions about the relationship between local feature perturbations and their impact on the model’s predictions. As such, other feature importance methods, such as Boruta-SHAP^38^ might find different relationships than LIME did.

## DISCUSSION

PAN-cODE offers competitive performance with several advantages over existing methods. While PAN-cODE uses a dynamical model for disease transmission, the actual dynamical function is learned directly from data, avoiding the need to manually specify an ODE function as in traditional compartmental models like the SIR model. However, it would be straightforward to incorporate a SIR compartmental model into PAN-cODE. By using the Neural ODE, PAN-cODE would be able to fit the dynamical parameters of this SIR model using backpropagation, avoiding the need for sampling or manual parameter specification in, eg, JHU_IDD-CovidSP.^39^ PAN-cODE also uses fewer data features compared to other methods such as Google_Harvard-CPF.^15^ Notably, PAN-cODE does not require mobility or hospitalization data, enabling forecasts in regions where data is limited, or at smaller regional resolutions. Due to the continuous-time Latent ODE architecture, PAN-cODE is also capable of natively handling datasets for pandemics where observations are sparsely or irregularly observed.

PAN-cODE directly learns the correlation between NPI stringency and future caseload without expert input or other manual adjustments. Consequently, the capability for PAN-cODE to provide alternative forecasts can be useful for policy-makers when determining the appropriate level of social restrictions required to obtain the desired level of transmission reduction. In contrast, existing methods capable of alternative outcome forecasting typically rely on manual adjustment of the R0 value in their compartmental model formulation.^39^^,^^40^ Historically, the process of manual estimation of pandemic trajectory can be error-prone.^41^ Given this complex relationship between NPI adoption and future caseload trajectories, PAN-cODE’s data-driven nature allows our capability to model this relationship to scale with the amount of available data. However, we do note that PAN-cODE does not explicitly learn a causal model between NPI stringency and future caseload. Building a formal causal model is likely difficult due to delayed and noisy reporting, and we leave this as future work.

Ideally, the counterfactuals in Figure 3 would show the effects of individual NPIs. Although we use individual NPIs to help predict the initial latent state, besides this, PAN-cODE’s predictions are only conditioned on the 4 stringency measures, because we found that the data on the individual NPIs was not rich enough to support a more precise conditioning. Richer data could be used to train a PAN-cODE model with the ability to model more counterfactuals.

## CONCLUSION

We present PAN-cODE, a deep conditional generative approach to pandemic forecasting using COVID-19 as an example. PAN-cODE conditions forecasts on NPI adoption stringency and can generate alternative caseload forecasts for modified NPI adoption stringencies. We demonstrate the performance of PAN-cODE on US state and county caseload forecasting and find it performs significantly better than all existing methods^10^^,^^15^^,^^21^^,^^25^^,^^26^^,^^29^^,^^39^^,^^42–50^ on 6-week death forecasting from March 8, 2021 and never performs significantly worse than the best performing method in all other death forecasting evaluation categories. Compared to existing methods, PAN-cODE requires minimal data features, can provide longer-term forecasts, and does not require retraining to generalize forecasts to unseen regions. The fully data-driven nature of PAN-cODE offers a scalable solution to inform public health response for COVID-19 and future pandemic outbreaks.

## FUNDING

Resources used in preparing this research were provided, in part, by the Ontario Institute for Cancer Research, the Memorial Sloan Kettering Cancer Center, the National Institute for Health (National Cancer Institute Cancer Center Support Grant P30 CA008748), the Province of Ontario, the Government of Canada through CIFAR, and companies sponsoring the Vector Institute www.vectorinstitute.ai/partners.

## AUTHOR CONTRIBUTIONS

RS and HZ implemented the method and experimental pipelines. All authors contributed toward development of the method and writing the manuscript.

## CONFLICT OF INTEREST STATEMENT

None declared.

## Supplementary Material

ocac160_Supplementary_Data

## Data Availability

Data on government NPI adoption were obtained from the Oxford COVID-19 Government Response Tracker^34^ and are available at: https://github.com/OxCGRT/covid-policy-tracker. All other data used in this article were obtained from Google Cloud Platform’s COVID-19 Open-Data^23^ repository and are available at: https://github.com/GoogleCloudPlatform/covid-19-open-data.
