# Supplementary material for: PAN-cODE: COVID-19 forecasting using conditional latent ODEs
Source: J Am Med Inform Assoc. 2022 Sep 1;29(12):2089–95. doi: 10.1093/jamia/ocac160 (PMC9667190; doi:10.1093/jamia/ocac160)
Supplement: ocac160_Supplementary_Data [file ocac160_supplementary_data.docx]

**SUPPLEMENTARY MATERIAL**

**Appendix A**

In the table below, we summarize the capability differences between PAN-cODE and benchmark methods from the COVID-19 Forecast Hub [21] that have an associated research article. The column “Manual Counterfactual” indicates that it is possible to generate alternative outcome trajectories through manual modification of the R0 value in a compartmental model. In contrast, “Data-Driven Counterfactual” models directly learn alternative outcome trajectories from data.

| **Method** | **Reference** | **Counterfactual**  **Possible** | **Manual**  **Counterfactual** | **Data-Driven Counterfactual** | **Requires Mobility Data** | **Requires Hospitalization Data** |
| --- | --- | --- | --- | --- | --- | --- |
| **PAN-cODE** | Ours | ✔ | ✘ | ✔ | ✘ | ✘ |
| **UMass-MechBayes** | [26] | ✔ | ✔ | ✘ | ✘ | ✔ |
| **CU-select** | [25] | ✔ | ✔ | ✘ | ✔ | ✔ |
| **COVIDhub-ensemble** | [21] | ✘ | ✘ | ✘ | ✔ | ✔ |
| **Caltech-CS156** | [42] | ✘ | ✘ | ✘ | ✔ | ✔ |
| **GT-DeepCOVID** | [43] | ✘ | ✘ | ✘ | ✔ | ✔ |
| **UCSD_NEU-DeepGLEAM** | [29] | ✘ | ✘ | ✘ | ✔ | ✘ |
| **Google_Harvard-CPF** | [15] | ✔ | ✔ | ✔ | ✔ | ✔ |
| **USC-SI_kJalpha** | [10] | ✔ | ✔ | ✘ | ✘ | ✘ |
| **UA-EpiCovDA** | [44] | ✔ | ✔ | ✘ | ✘ | ✘ |
| **JHU_IDD-CovidSP** | [38] | ✔ | ✔ | ✘ | ✔ | ✔ |
| **Columbia_UNC-SurvCon** | [45] | ✔ | ✔ | ✘ | ✘ | ✘ |
| **UCLA-SuEIR** | [46] | ✔ | ✔ | ✘ | ✘ | ✘ |
| **TTU-squider** | [47] | ✔ | ✔ | ✘ | ✘ | ✘ |
| **IowaStateLW-STEM** | [48] | ✔ | ✔ | ✘ | ✔ | ✘ |
| **JHUAPL-Bucky** | [49] | ✔ | ✔ | ✘ | ✔ | ✔ |
| **Covid19Sim-Simulator** | [50] | ✔ | ✔ | ✘ | ✘ | ✔ |

In the table below, we summarize the mechanism of the above methods, two of our baselines, and the negative control baseline of re-using the previous week’s count.

| **Method** | **Reference** | **Description** |
| --- | --- | --- |
| **UMass-MechBayes** | [26] | Models SEIR parameters as probability distributions. Trained with HMC. |
| **CU-select** | [25] | SEIR model incorporated into a stochastic process model with improved transmission model. |
| **COVIDhub-ensemble** | [21] | Ensemble of models submitted to the COVID-19 Forecast Hub |
| **Caltech-CS156** | [42] | Ensemble of many deep learning, statistical, and compartmental models. |
| **GT-DeepCOVID** | [43] | Feedforward network with autoregressive inputs. |
| **UCSD_NEU-DeepGLEAM** | [29] | Graph CNN over 25km by 25km spatial cells. |
| **Google_Harvard-CPF** | [15] | Models SEIR parameters as time-varying functions of covariates. |
| **USC-SI_kJalpha** | [10] | Augmenting compartmental models with cross-region transmission. |
| **UA-EpiCovDA** | [44] | Estimates SIR parameters analytically from an incidence vs. cumulative cases curve. |
| **JHU_IDD-CovidSP** | [38] | Probabilistic compartmental model with mobility matrix between meta-populations. |
| **Columbia_UNC-SurvCon** | [45] | Dynamic model with time-varying transmission rate. |
| **UCLA-SuEIR** | [46] | Compartmental model with undetected cases. Parameters fit by gradient descent against log MSE loss. |
| **TTU-squider** | [47] | Compartmental model with power-law incidence rate. |
| **IowaStateLW-STEM** | [48] | Nonparametric space-time disease transmission model estimated with a quasi-likelihood approach. |
| **JHUAPL-Bucky** | [49] | County level SEIR model which incorporates multiple covariates. Parameters estimated through Monte Carlo simulation |
| **Covid19Sim-Simulator** | [50] | SEIR model which incorporates hospitalization and vaccination rates. Parameters fit using simulated annealing. |
| **PrevWeek** | - | Predict weekly deaths or infections to be the same as the last week with observed data. |
| **VAE-GRU** | Ours | Variational network with a GRU encoder and decoder. Decoder only predicts forward. |
| **GRU-ODE** | Ours | GRU with hidden states modelled using a Neural ODE between observations. |

**Appendix B**

In the table below, we provide the evaluation of PAN-cODE on infection count prediction. We show median absolute error (MAE) of the forecasted total infections for PAN-cODE versus several baselines for all states of the United States. We also show the mean rank of each method across all states, in comparison to other baselines. Results that are not significantly greater than the best model in the category (Wilcoxon sign-rank P > 0.05) are shown in bold italics. The best-performing method evaluated by each metric is underlined.

|  | **2020-12-28 Forecast** | | | | **2021-03-08 Forecast** | | | |
| --- | --- | --- | --- | --- | --- | --- | --- | --- |
|  | **4 Weeks Ahead** | | **6 Weeks Ahead** | | **4 Weeks Ahead** | | **6 Weeks Ahead** | |
| **Model** | **MAE** | **Mean  Rank** | **MAE** | **Mean Rank** | **MAE** | **Mean Rank** | **MAE** | **Mean Rank** |
| **PAN-cODE** | ***10,248*** | 6.14 | 16,317 | 5.1 | 4,465 | 8.55 | 8,230 | 7.9 |
| **IowaStateLW-STEM** | ***8,049*** | ***5.12*** | 18,334 | 5.02 | 3,656 | 6.73 | 7,330 | 5.37 |
| **JHU_IDD-CovidSP** | 19,974 | 8.08 | ***19,054*** | ***4.73*** | 4,529 | 9.04 | 6,399 | 7.04 |
| **Covid19Sim-Simulator** | ***8,688*** | ***4.69*** | ***8,227*** | ***3.53*** | 3,206 | 6.98 | 6,463 | 5.92 |
| **UCLA-SuEIR** | 13,537 | 8.25 | 16,933 | 5.43 | 10,551 | 10.8 | 16,149 | 8.37 |
| **CU-select** | 25,485 | 10.43 | 27,384 | 6.82 | ***4,106*** | ***7.49*** | ***8,205*** | ***5.63*** |
| **USC-SI_kJalpha** | ***6,962*** | 6.04 | - | - | ***3,605*** | ***5.69*** | 5,694 | 5.27 |
| **JHUAPL-Bucky** | 31,616 | 10.88 | 42,185 | 7.69 | 5,913 | 9.39 | 10,084 | 8.1 |
| **GRU-ODE** | 63,010 | 14.22 | 82,095 | 10.43 | 9,044 | 14 | 13,295 | 11.2 |
| **VAE-GRU** | 63,795 | 14.08 | 77,953 | 9.78 | 9,600 | 12.96 | 14,779 | 10.57 |
| **COVIDhub-ensemble** | ***8,920*** | 5.75 | - | - | 2,991 | 5.96 | - | - |
| **Google_Harvard-CPF** | 12,472 | 6.94 | - | - | 3,587 | 7.49 | - | - |
| **TTU-squider** | 21,768 | 8.86 | - | - | 5,952 | 10.45 | - | - |
| **Mean COVID Hub** | 14,021 | - | 19,041 | - | 4,629 | - | 8,108 | - |
| **Baseline (PrevWeek)** | ***9,020*** | ***5.26*** | ***13499*** | ***3.74*** | ***2,684*** | ***5.5*** | ***4,870*** | ***4.21*** |

**Appendix C**

PAN-cODE uses a 14-day shift between caseload trajectories and covariate data features and applies a seven-day rolling mean to the caseload trajectories before input. This appendix documents the performance of alternative choices. We note that the choice to use a 14-day shift and seven-day rolling mean was selected using validation performance. The test results shown below did not influence hyperparameter selection. In the tables below, we show the performance as measured by mean squared error (MAE) and root mean squared error (RMSE) of the PAN-cODE architecture using alternative shifts. Results that are not significantly greater than the best model in the category (Wilcoxon sign-rank P > 0.05) are shown in bold italics.

| **Infection Prediction** | **2020-12-28 Forecast** | | | | **2021-03-08 Forecast** | | | |
| --- | --- | --- | --- | --- | --- | --- | --- | --- |
|  | **4 Weeks Ahead** | | **6 Weeks Ahead** | | **4 Weeks Ahead** | | **6 Weeks Ahead** | |
| **Model** | **MAE** | **RMSE** | **MAE** | **RMSE** | **MAE** | **RMSE** | **MAE** | **RMSE** |
| **PAN-cODE (14-day Shift)** | ***25,687*** | ***51,921*** | ***40,303*** | ***83,552*** | 10,607 | 19,546 | 23,794 | ***43,225*** |
| **6-day Shift** | 36,339 | 79,159 | 54,371 | 117,268 | ***9,631*** | ***19,444*** | ***22,263*** | ***42,824*** |
| **10-day Shift** | 87,911 | 160,173 | 122,683 | 219,917 | 13,809 | 25,988 | 28,985 | 51,877 |
| **18-day Shift** | 84,897 | 154,966 | 118,495 | 212,935 | 20,072 | 26,622 | 36,263 | 64,189 |
| **22-day Shift** | 53,971 | 103,357 | 73,962 | 138,158 | 16,026 | 29,150 | 31,337 | 55,090 |

| **Death Prediction** | **2020-12-28 Forecast** | | | | **2021-03-08 Forecast** | | | |
| --- | --- | --- | --- | --- | --- | --- | --- | --- |
|  | **4 Weeks Ahead** | | **6 Weeks Ahead** | | **4 Weeks Ahead** | | **6 Weeks Ahead** | |
| **Model** | **MAE** | **RMSE** | **MAE** | **RMSE** | **MAE** | **RMSE** | **MAE** | **RMSE** |
| **PAN-cODE (14-day Shift)** | ***380*** | ***831*** | ***644*** | ***1,511*** | ***177*** | ***322*** | ***249*** | ***494*** |
| **6-day Shift** | 798 | 1599 | 1,263 | 2,614 | ***239*** | ***538*** | 354 | ***751*** |
| **10-day Shift** | 1,436 | 2,472 | 2,251 | 3,911 | ***197*** | ***323*** | 343 | 556 |
| **18-day Shift** | 1,301 | 2,290 | 2,095 | 3,620 | 325 | 744 | 511 | 1,061 |
| **22-day Shift** | 1,257 | 1,599 | 2,003 | 3,620 | ***253*** | ***567*** | ***370*** | ***778*** |

The performance of PAN-cODE using alternative data smoothing functions is similarly shown below. We apply a 14-day rolling mean (RM14), a 7-day exponentially weighted moving average (EWMA), and a Savitzky-Golay filter (SG) with period of 31 and polynomial order of 5.

| **Infection Prediction** | **2020-12-28 Forecast** | | | | **2021-03-08 Forecast** | | | |
| --- | --- | --- | --- | --- | --- | --- | --- | --- |
|  | **4 Weeks Ahead** | | **6 Weeks Ahead** | | **4 Weeks Ahead** | | **6 Weeks Ahead** | |
| **Model** | **MAE** | **RMSE** | **MAE** | **RMSE** | **MAE** | **RMSE** | **MAE** | **RMSE** |
| **PAN-cODE (RM7)** | ***25,676*** | ***51,911*** | ***40,283*** | ***83,540*** | ***10,607*** | ***19,546*** | 23,794 | 43,225 |
| **PAN-cODE (RM14)** | 51,829 | 85,115 | 62,149 | 115,899 | 13,847 | 26,022 | 27,807 | 50,194 |
| **PAN-cODE (EWMA)** | 45,701 | 113,447 | 72,123 | 161,823 | ***12,167*** | ***23,798*** | ***20,653*** | ***41,116*** |
| **PAN-cODE (SG)** | 90,862 | 176,339 | 124,973 | 236,433 | 31,285 | 51,364 | 49,736 | 81,059 |

| **Death Prediction** | **2020-12-28 Forecast** | | | | **2021-03-08 Forecast** | | | |
| --- | --- | --- | --- | --- | --- | --- | --- | --- |
|  | **4 Weeks Ahead** | | **6 Weeks Ahead** | | **4 Weeks Ahead** | | **6 Weeks Ahead** | |
| **Model** | **MAE** | **RMSE** | **MAE** | **RMSE** | **MAE** | **RMSE** | **MAE** | **RMSE** |
| **PAN-cODE (RM7)** | ***380*** | ***831*** | ***644*** | ***1,511*** | ***177*** | ***322*** | ***249*** | ***494*** |
| **PAN-cODE (RM14)** | 865 | 1,636 | 1,204 | 2,126 | ***194*** | ***401*** | ***340*** | 678 |
| **PAN-cODE (EWMA)** | 938 | 2,086 | 1,538 | 3,379 | ***195*** | ***486*** | 308 | 655 |
| **PAN-cODE (SG)** | 759 | 1,548 | 1,204 | 2,126 | 507 | 950 | 702 | 1276 |

Overall, using a 14-day shift and seven-day rolling mean also results in the best test performance.

Additional data modalities such as the Google Community Mobility Reports (GCMR) [23] can be easily provided to PAN-cODE. The GCMR tracks human mobility in six community locations, such as workplaces, residential areas, or retail stores, as a percentage change in comparison to a pre-COVID baseline. These values are provided at the state and county level, but data is often missing due to privacy concerns. Below, we report an experiment where GCMR mobility data is included as a covariate. Missing data points were imputed using values from their parent geographic region. For example, if a county missing data for a specific day, the value is imputed from the state the county exists in. Results are shown below.

| **Infection Prediction** | **2020-12-28 Forecast** | | | | **2021-03-08 Forecast** | | | |
| --- | --- | --- | --- | --- | --- | --- | --- | --- |
|  | **4 Weeks Ahead** | | **6 Weeks Ahead** | | **4 Weeks Ahead** | | **6 Weeks Ahead** | |
| **Model** | **MAE** | **RMSE** | **MAE** | **RMSE** | **MAE** | **RMSE** | **MAE** | **RMSE** |
| **PAN-cODE** | ***25,676*** | ***51,911*** | ***40,283*** | ***83,540*** | ***10,607*** | ***19,546*** | 23,794 | 43,225 |
| **+ Mobility Data** | 85,836 | 148,112 | 121,665 | 208,976 | 16,816 | 29,768 | 32,625 | 56,583 |

| **Death Prediction** | **2020-12-28 Forecast** | | | | **2021-03-08 Forecast** | | | |
| --- | --- | --- | --- | --- | --- | --- | --- | --- |
|  | **4 Weeks Ahead** | | **6 Weeks Ahead** | | **4 Weeks Ahead** | | **6 Weeks Ahead** | |
| **Model** | **MAE** | **RMSE** | **MAE** | **RMSE** | **MAE** | **RMSE** | **MAE** | **RMSE** |
| **PAN-cODE** | ***380*** | ***831*** | ***644*** | ***1,511*** | ***177*** | ***322*** | ***249*** | ***494*** |
| **+ Mobility Data** | 1, 303 | 2,247 | 2,084 | 3,646 | 288 | 605 | 486 | 925 |

While the inclusion of mobility data should improve performance, we found that PAN-cODE performed worse. This could be caused by the data imputation method, which may cause the model to learn false relationships between mobility and caseload.

**Appendix D**

Here, we report the architectural and training hyper-parameters used for the final PAN-cODE model. Due to varying data cut-offs, we trained two separate models for each forecasting date.

PAN-cODE was trained using the Adam optimizer. We reduce the learning rate by a factor of 0.1 after a plateau of 10 epochs without improvement in prediction loss. After a minimum number of epochs, we stop training when loss does not improve after 20 epochs. The Neural ODE in the GRU-ODE and Latent ODE uses identical architectures, with the number of layers and units representing the dynamical function varying per model. We multiple samples from the variational posterior to compute the gradient for each iteration. We tune for the fixed variance used to compute the likelihood in the ELBO. We also attempted to use an importance-weighted ELBO but found it did not improve results.

After an initial manual search of suitable hyper-parameters, we performed a grid search on plausible ranges of hyper-parameters. Grid values are reported in the table below. We select each final model using the loss on the validation prediction region, which is the month before the true forecasting date, held out from the training data.

| **Hyper-parameter** | **Grid Values** | **2020-12-28** | **2021-03-08** |
| --- | --- | --- | --- |
| **Batch size** | [32, 256, 1024] | 256 | 32 |
| **Initial learning rate** | [1e-3, 1e-4] | 1e-3 | 1e-3 |
| **Neural ODE layers** | [3, 4] | 3 | 3 |
| **Neural ODE units** | [128, 250, 500] | 128 | 128 |
| **Latent dimension** | [32] | 32 | 32 |
| **Dropout** | [0, 0.25] | 0.25 | 0.25 |
| **Minimum epochs** | [150, 250, 1000] | 250 | 250 |
| **Latent samples** | [32, 50] | 50 | 50 |
| **ELBO fixed variance** | [0.1, 0.5, 1] | 0.5 | 0.1 |

**Appendix E**

Feature importance methods such as LIME [37] can be applied to PAN-cODE to identify key features driving its predictions. LIME generates perturbed inputs for a specific forecast and uses the model of interest (PAN-cODE) to generate output given these perturbed inputs. LIME then fits a locally linear model to the feature perturbations and the outputs of PAN-cODE, and the coefficients of this linear model correspond to the feature importance estimates.

Below, we apply LIME to the infection forecasts of Fresno County, CA. Two experiments are run for two dates, 21 days and 10 days before the forecasting date. The two figures below show the coefficients fit by LIME. The stringency_index_norm feature was removed due to colinearity with other NPI indices. The random input perturbation performed by LIME is repeated 50 times to generate the boxplots shown in the figure below. Features whose mean coefficient values are statistically significantly different than zero (t-test P>0.05) are shown in bold. Green boxes indicate a positive correlation between the feature and the overall number of infections in the prediction region, and red boxes indicate a negative correlation.

In the figures below, we see that adopting preventative measures has a high impact on infection counts earlier in the encoding region, but nearer to the forecasting date, the actual caseload is more impactful. This behaviour is consistent with the known lagged effect of NPIs.


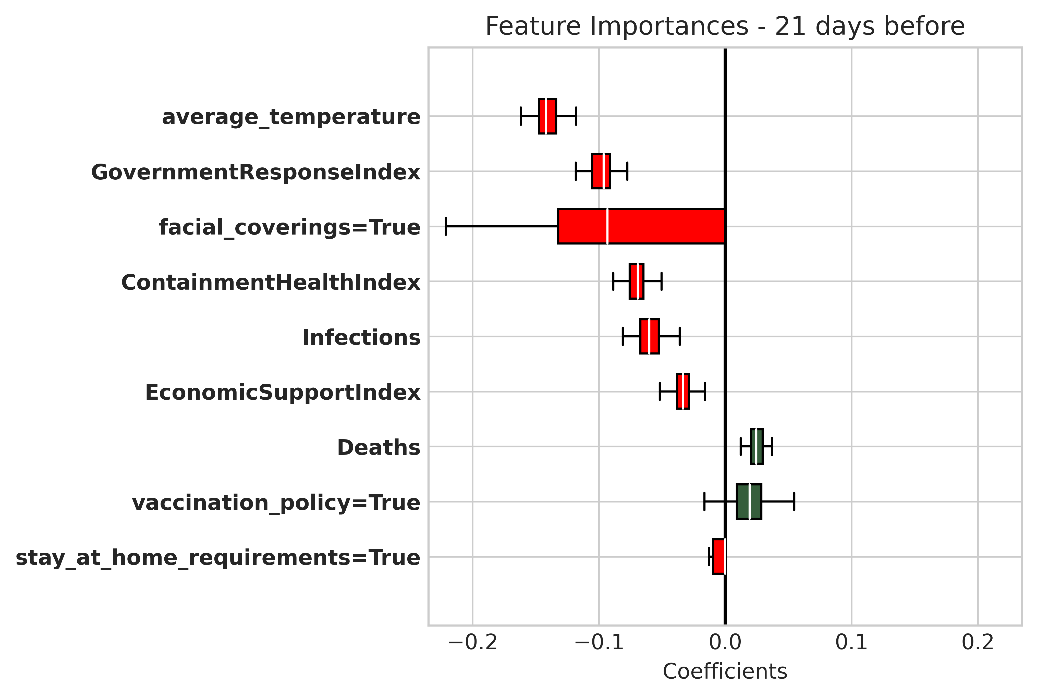


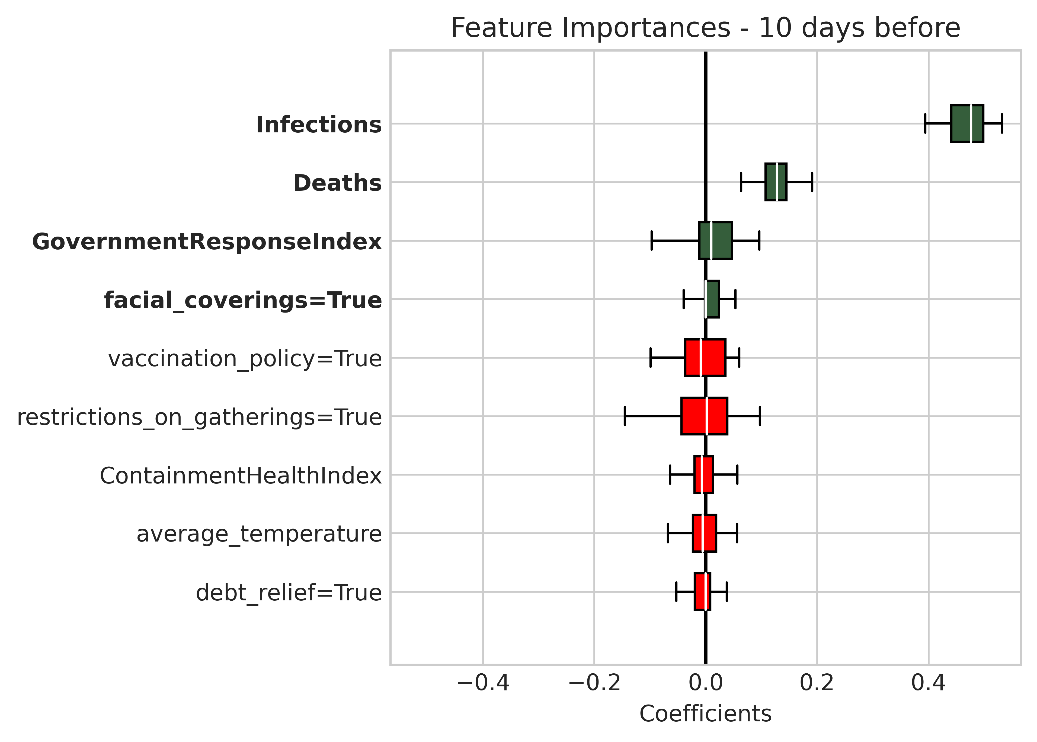


The features shown in the above figure are derived from the previously referenced Google and OxGCRT datasets. In the table below, we provide a short summary of the features. Detailed descriptions are available at the original data source repositories, which can be found in the data disclosure.

| **Feature Name** | **Description** |
| --- | --- |
| Infections | The daily increase in COVID-19 infections. |
| Deaths | The daily increase in COVID-19 deaths. |
| average_temperature | Daily mean temperature. |
| GovernmentResponseIndex | OxGCRT aggregate metric which tracks the overall level of policy response to COVID-19 |
| ContainmentHealthIndex | OxGCRT aggregate metric which tracks the policy response that specifically aim to contain COVID-19 spread and support health systems. |
| EconomicSupportIndex | OxGCRT aggregate metric which track policys aimed to mitigate the economic impact of COVID-19. |
| facial_coverings | Binary variable which tracks whether facial coverings are mandated. |
| vaccination_policy | Binary variable which tracks whether vaccines are available to the population. |
| stay_at_home_requirements | Binary variable which tracks whether any stay at home mandates are active. |
| restrictions_on_gatherings | Binary variable which tracks whether any restrictions on gatherings are active. |
| debt_relief | Binary variable which tracks whether debt relief policies are active. |
